# Parental Perception Towards Flu Vaccination for Asthmatic Children in Saudi Arabia

**DOI:** 10.7759/cureus.6460

**Published:** 2019-12-25

**Authors:** Alaa AlQurashi, Hala Aljishi, Enass Demyati

**Affiliations:** 1 Epidemiology and Public Health, King Fahad Medical City, Riyadh, SAU; 2 Family Medicine, King Fahad Medical City, Riyadh, SAU

**Keywords:** asthma, flu vaccination, children

## Abstract

Background

Asthma is a major noncommunicable disease that affects around 235 million people, including children, globally. In the Kingdom of Saudi Arabia (KSA), the incidence of childhood asthma continues to increase. The Ministry of Health in Saudi Arabia has attempted to put policies in place to prevent the occurrence of asthma-related complications by encouraging parents to vaccinate their diagnosed asthmatic children with the flu vaccine. To date, however, there have been no studies investigating the use of flu vaccine among asthmatic children in KSA. Our research aims to explore the perception of parents with asthmatic children towards flu vaccination and its effect on the decision to vaccinate their children.

Methods

Our research was a cross-sectional study of 190 parents who presented with asthmatic children at King Fahad Medical City (KFMC) in Riyadh, KSA from October 2016 to April 2017. The study tools included structured and semi-structured questionnaires with demographic information, types of healthcare, and perceptions of parents towards flu vaccination. Data analysis was done using Statistical Package for the Social Sciences (SPSS; IBM, Armonk, NY).

Results

Samples were mostly Saudis (97%), who were married (92%), and in the age group of 21-40 years (70%). Most of them were females (59%). More than two-thirds of the parents had a middle school education or bachelor's degree, and more than half were employed. Parents with higher education had a higher rate of vaccination for their children, and they were more likely to believe that a non-vaccinated child is more likely to get flu. Almost 76% of parents with vaccinated children agreed that the flu vaccine could safeguard children against flu. Doctors' opinion about flu vaccination was significantly associated with the parents' decision. The multivariate regression analysis results showed that easy access to services and parents’ beliefs regarding vaccination are positively associated with influenza vaccination status.

Conclusion

Among the essential factors positively associated with the influenza vaccination status were a perception of easy access to vaccination services and the belief that non-vaccinated children are more likely to contract the flu virus. In contrast, the belief that vaccination prevents infection by the flu virus was negatively associated with vaccine uptake.

## Introduction

Asthma, a major non-communicable disease characterized by recurrent attacks of breathlessness and wheezing, affects around 235 million people globally. The World Health Organization (WHO) has reported ann estimated 383,000 asthma-related deaths in 2015, many of which occurred in low- and middle-income countries [1]. It is a common disease among children. While asthma may be triggered by numerous factors (e.g., polluted air, outdoor and indoor allergens, and stressful lifestyle), viral infection of the respiratory tract (e.g., influenza) remains the leading risk factor for childhood asthma [2]. Studies have shown that approximately 85% of asthma attacks in children are related to this viral infection, which exacerbates asthma symptoms and leads to admission to the emergency department and hospitalization [3,4,5].

In the Kingdom of Saudi Arabia (KSA), the incidence of childhood asthma continues to increase [6]. The American Academy of Pediatrics and the WHO have recommended an annual administration of influenza vaccination for asthmatic children during fall and winter seasons [7,8]. These vaccinations are recommended as a precautionary measure to control serious asthma complications, including pneumonia [8]. Numerous research studies have been conducted to investigate the barriers and facilitators for vaccine uptake in the US [9]. However, despite these recommendations, a low rate of adherence to vaccination guidelines has been reported in many countries. Notably, the Middle Eastern countries account for only 3.7% of the flu vaccines administered worldwide [10].

Several studies have investigated the factors contributing to higher asthma prevalence among children [11,12]. In addition to poor air quality and environmental factors, viral infections are the most common triggering factor that leads to asthma in children aged 4 to 10 years [13]. The Saudi Ministry of Health has devised a management plan to control the occurrence of asthma-related complications [14]. The program focuses on raising public awareness by educating and encouraging parents to inoculate their asthmatic children with the flu vaccine. To date, however, there have been no studies investigating the details of the administration of flu vaccine among asthmatic children in KSA. Most of the current studies explore the adherence of healthcare workers to the recommendations for flu vaccination [15]. Thus, our research aims to examine the perception of parents with asthmatic children toward flu vaccination and its association with their decision to vaccinate their children in KSA.

## Materials and methods

This cross-sectional study was conducted at the King Fahad Medical City (KFMC) in Riyadh, KSA, from September 2016 to April 2017. KFMC is an academic teaching hospital with a capacity of 600 beds, catering to patients in Riyadh and the surrounding areas. We interviewed a total of 190 parents at the family medicine clinic and asthma clinic. The inclusion criteria included: 1) parents who had at least one asthmatic children aged 6 months to 15 years; 2) parents of children who were admitted to the emergency department and/or had been hospitalized during the previous 12 months; and 3) parents of children receiving medications for asthma (i.e., inhaled steroid, oral steroid, leukotriene antagonist, cromolyn/nedocromil, and salmeterol or theophylline). All clinical information was obtained from the electronic medical records of KFMC. Parents of children without a medical record in KFMC and those of children with medical conditions considered as contraindications to vaccination against the flu were excluded.

In terms of the tool we used, this study is similar to a previous study conducted in the US, which examined modifiable factors contributing to increased vaccination rates [16]. The survey tool was adopted from the Triandis model [16]. The original self-report instrument was designed to collect information regarding the facilitating conditions, behavioral habits, value of consequences of the activity, social influences (including the influence of the clinician on patients), and attitudes toward the activity [16]. This model has been used and validated in numerous settings for the investigation of health-related behaviors [17,18]. The survey tool was translated into Arabic, validated using a forward-back translation by two independent translators, and piloted on 10 participants to ensure the validity of the results. The semi-structured questionnaire included demographic and statements on knowledge and attitudes, perceived benefits, social support, adverse events observed, and perceived barriers to adherence. Each category was based on the Likert agreement scale and used to measure the respondents’ agreement. All data were analyzed using the statistical package SPSS version 22.0 (IBM, Armonk, NY).

In terms of sampling, a trained research assistant approached all parents attending the family medicine clinic and asthma clinic each week from October 2016 to April 2017. Parents who agreed to participate in the study and signed the informed consent form were interviewed. The response rate was 100%, and all invited participants completed the questionnaire. The sample was conveniently collected to include all eligible parents attending the flu and family clinics each week. The asthma clinic was included to limit any possible participant-selection bias of parents attending it not being aware of the availability of the flu vaccine. Recall bias regarding vaccination data was controlled by rechecking the hospital records to confirm the flu vaccination status.

In terms of data analysis, all categorical variables such as sex, educational level, and vaccination status are presented as numbers and percentages. Based on whether the cell expected frequency was <5, the chi-squared or Fisher’s exact test was used to determine significant relationships between categorical variables. Binary logistic regression was applied to determine the significant factors associated with parental perception regarding the flu vaccine. A p-value below 0.05 denoted a statistically significant difference. Ethical approval was obtained from the Institution Review Board at KFMC. 

## Results

A total of 190 of Saudi parents were interviewed. An overwhelming majority of them were Saudis nationals (97%). Most of the interviewed parents were aged 20-40 years. Almost 60% of the interviewed parents were mothers. Of note, 88 parents were vaccinated (46.3%), whereas 102 parents were not (53.7%) (Table 1).

**Table 1:** Descriptive statistics of sample characteristics and parental perceptions N: number of respondents

Characteristics (n = 190)	Description	N (%)
(a) Sample characteristics		
Nationality	Saudi	185 (97.4%)
	Non-Saudi	5 (2.6%)
Age	≤20	14 (7.4%)
	21–40	133 (70%)
	>40	43 (22.6%)
Sex	Male	78 (41.1%)
	Female	112 (58.9%)
Marital status	Married	174 (91.6%)
	Divorced	2 (1.1%)
	Other	14 (7.4%)
Educational level	Not educated	10 (5.3%)
	Middle education	68 (35.8%)
	Bachelor	73 (38.4%)
	Higher education	39 (20.5%)
Occupation	Employed	100 (52.6%)
	Unemployed	78 (41.1%)
	Other	12 (6.3%)
Asthmatic children flu vaccination status	Vaccinated	88 (46.3%)
	Non-vaccinated	102 (53.7%)
(b) Perceptions		
Children with asthma should receive flu vaccination	Agree	173 (91.1%)
	Do not agree	4 (2.1%)
	Do not know	13 (6.8%)
I received a reminder from the primary care unit/hospital regarding my child’s flu vaccination appointment	Agree	137 (72.1%)
	Do not agree	25 (13.2%)
	Do not know	28 (14.7%)
It is easy to reach the primary care unit/hospital to receive the flu vaccination	Agree	166 (87.4%)
	Do not agree	14 (7.4%)
	Do not know	10 (5.3%)
I believe that my child has to receive the flu vaccination	Agree	173 (91.1%)
	Do not agree	8 (4.2%)
	Do not know	9 (4.7%)
The flu vaccination may cause complications/troubles to my child	Agree	36 (18.9%)
	Do not agree	122 (64.2%)
	Do not know	32 (16.8%)
The maximum limit of flu vaccine for children per visit is	One shot	108 (56.8%)
	Two shots	82 (43.2%)
	Three shots	0 (0.0%)
	Four shots	0 (0.0%)
I believe that my child is sick because of the flu shot	Agree	53 (27.9%)
	Do not agree	113 (59.5%)
	Do not know	24 (12.6%)
Comparing the number of times that my child got sick during last year’s winter versus this winter	Less than last winter	99 (52.1%)
	More than last winter	50 (26.3%)
	No difference	41 (21.6%)
My child’s pediatrician believes that my child should receive the flu vaccine	Agree	166 (87.4%)
	Do not agree	10 (5.3%)
	Do not know	14 (7.4%)
My relatives believe that my child should receive the flu vaccine	Agree	144 (75.8%)
	Do not agree	19 (10.0%)
	Do not know	27 (14.2%)
Non-vaccinated children are more likely to contract the flu	Agree	117 (61.6%)
	Do not agree	50 (26.3%)
	Do not know	23 (12.1%)
The flu vaccination prevents infection by the flu virus	Agree	157 (82.6%)
	Do not agree	12 (6.3%)
	Do not know	21 (11.1%)
I am worried about the chances of my child contracting the flu because of the flu vaccine	Agree	45 (23.7%)
	Do not agree	119 (62.6%)
	Do not know	26 (13.7%)

Table 2 shows the association between parental characteristics and children’s vaccination status. In terms of sociodemographic parameters, the results show that a higher education level was positively associated with vaccination uptake among asthmatic children (p-value = 0.042 and <0.001, respectively). Parents aged 21-40 years were more likely to vaccinate their children compared with those aged >40 years (p-value = <0.001). In terms of knowledge and facilitating conditions, a large number of parents with vaccinated children agreed that non-vaccinated children are more likely to contract the flu (p-value = <0.001). Unexpectedly, parents of both vaccinated and non-vaccinated children reported that children with asthma should receive the flu vaccine (p-value = 0.002). Moreover, they reported that their pediatrician had recommended that the children should receive the flu vaccine (p-value = 0.016), and the hospital had made the vaccine accessible to the parents (p-value = 0.002).

**Table 2:** Association between parental characteristics and vaccination status *Significant p-value

Characteristic of the parents	Description	Vaccinated	Non-vaccinated	P-value
Educational level	Not educated	2 (2.3%)	8 (7.8%)	<0.001*
	Middle education	20 (22.7%)	48 (47.1%)	
	Bachelor	30 (34.1%)	43 (42.2%)	
	Higher education	36 (40.9%)	3 (2.9%)	
Age	≤20	13 (14.8%)	1 (1.0%)	<0.001*
	21–40	46 (52.3%)	87 (85.3%)	
	>40	29 (33%)	14 (13.7%)	
Sex	Male	35 (39.8%)	43 (42.2%)	0.739
	Female	53 (60.2%)	59 (57.8%)	
Nationality	Saudi	87 (98.9%)	98 (96.1%)	0.232
	Non-Saudi	1 (1.1%)	4 (3.9%)	
Social status	Married	76 (86.4%)	98 (96.1%)	0.042*
	Divorced	1 (1.1%)	1 (1.0%)	
	Other	11 (12.5%)	3 (2.9%)	
Occupation	Employed	39 (44.3%)	61 (59.8%)	0.098
	Unemployed	43 (48.9%)	35 (34.3%)	
	Other	6 (6.8%)	6 (5.9%)	
Children with asthma should receive the flu vaccination	Agree	77 (87.5%)	96 (94.1%)	0.224
	Do not agree	2 (2.3%)	2 (2.0%)	
	Do not know	9 (10.2%)	4 (3.9%)	
I received a reminder from the primary care unit/hospital regarding my child’s flu vaccination appointment	Agree	64 (72.7%)	73 (71.6%)	0.641
	Do not agree	13 (14.8%)	12 (11.8%)	
	Do not know	11 (12.5%)	17 (16.7%)	
It is easy to reach the primary care unit/hospital to receive the flu vaccination	Agree	69 (78.4%)	97 (95.1%)	0.002*
	Do not agree	12 (13.6%)	2 (2.0%)	
	Do not know	7 (8.0%)	3 (2.9%)	
I believe that my child has to receive the flu vaccination	Agree	78 (88.6%)	95 (93.1%)	0.533
	Do not agree	5 (5.7%)	3 (2.9%)	
	Do not know	5 (5.7%)	4 (3.9%)	
The flu vaccine may cause complications/troubles to my child	Agree	23 (26.1%)	13 (12.7%)	<0.001*
	Do not agree	42 (47.7%)	80 (78.4%)	
	Do not know	23 (26.1%)	9 (8.8%)	
The maximum limit of flu vaccine for children per visit is	One shot	83 (94.3%)	25 (24.5%)	<0.001*
	Two shots	5 (5.7%)	77 (75.5%)	
I believe that my child is sick because of the flu shot	Agree	29 (33.0%)	24 (23.5%)	0.266
	Do not agree	47 (53.4%)	66 (64.7%)	
	Do not know	12 (13.6%)	12 (11.8%)	
Comparing the number of times that my child got sick during last year’s winter versus this winter	Less than last winter	20 (22.7%)	79 (77.5%)	<0.001*
	More than last winter	46 (52.3%)	4 (3.9%)	
	No difference	22 (25.0%)	19 (18.6%)	
My child’s pediatrician believes that my child should receive the flu vaccine	Agree	72 (81.8%)	94 (92.2%)	0.016*
	Do not agree	9 (10.2%)	1 (1.0%)	
	Do not know	7 (8.0%)	7 (6.9%)	
My relatives believe that my child should receive the flu vaccine	Agree	64 (72.7%)	80 (78.4%)	0.340
	Do not agree	8 (9.1%)	11 (10.8%)	
	Do not know	16 (18.2%)	11 (10.8%)	
Non-vaccinated children are more likely to contract the flu	Agree	63 (71.6%)	54 (52.9%)	<0.001*
	Do not agree	10 (11.4%)	40 (39.2%)	
	Do not know	15 (17.0%)	8 (7.8%)	
The flue vaccination prevents infection by the flu virus	Agree	67 (76.1%)	90 (88.2%)	0.008*
	Do not agree	7 (8.0%)	5 (4.9%)	
	Do not know	14 (15.9%)	7 (6.9%)	
I am worried about the chances of my child contracting the flu because of the flu vaccine	Agree	31 (35.2%)	14 (13.7%)	0.002*
	Do not agree	46 (52.3%)	73 (71.6%)	
	Do not know	11 (12.5%)	15 (14.7%)	

In terms of attitudes, there was no significant association between attitudes and perceptions of parents toward vaccine uptake. A large number of parents (n = 78) agreed that a child should receive the flu vaccine, whereas only five disagreed; this belief was not reflected in the association with vaccine uptake. The attitude of parents toward vaccination (e.g., concerns regarding potential adverse effects, the required number of vaccine shots, and the comparison of flu episodes prior to and after receiving the vaccine) was significantly associated with flu vaccine uptake (p-value = <0.001). In terms of social support, physicians thought that vaccination against the flu was significantly associated with parents’ decision to vaccinate their children (p-value = 0.016). Parents with vaccinated children (81.8%) and those with non-vaccinated children (92.2%) agreed with the following statement: “my child’s pediatrician believes that my child should receive the flu vaccine.” In terms of perceived consequences, there was a significant association between the perceived consequences and flu vaccine uptake. The majority of parents (71%) reported that non-vaccinated children were more likely to contract the flu (p-value = <0.001). Almost 76% of the parents with vaccinated children agreed that the flu vaccine prevented infection by the flu virus (p-value = 0.008). Only 35% of the parents were worried about contracting the flu due to the vaccine (p-value = 0. 002).

Table 3 shows the independent factors associated with the uptake of the flu vaccine in children using multivariate logistic regression. Results show that the most important factors positively associated with the influenza vaccination status were easy access to vaccination services and the perception of parents that non-vaccinated children are more likely to contract the flu virus. In contrast, worrying about the chance of contracting the flu due to the vaccine and the belief that vaccination prevents infection by the flu virus were negatively associated with vaccine uptake.

**Table 3:** Independent factors associated with the flu vaccine uptake OR: odds ratio; CI: Confidence interval; *Reference range: do not know/do not agree **Significant p-value

Survey description	OR (95% CI)	P-value
It is easy to reach the primary care unit/hospital to receive the flu vaccination	0.206 (0.07–0.608)*	0.004**
Non-vaccinated children are more likely to contract the flu	2.479 (1.200–5.119)*	0.014**
The flu vaccination prevents infection by the flu	0.395 (0.157–0.992)*	0.048**
I am worried about the chances of my child contracting the flu because of the flu vaccine	2.913 (1.367–6.208)*	0.006**

## Discussion

Our findings show that parents' age and education were significantly associated with the children's vaccine uptake. These findings are similar to a cross-sectional study conducted in Taiwan, where 50% of the participating parents were females with at least a middle-school education [19]. Moreover, a web-based study by Flood et al. reported that the decision to vaccinate was related to parental knowledge on the importance of childhood vaccination [20]. Parents who were aware of the flu were more likely to vaccinate their children. However, in our study, the parental level of knowledge was not positively associated with vaccine uptake in children. A possible explanation is that other factors, such as access to a healthcare service provider, may affect the decision to administer the vaccine as well.

The facilitating conditions found to be associated with the rate of vaccination among asthmatic children in KSA were consistent with the results obtained from other studies. A study by Daley et al. reported that regular reminders increased the chances of parents vaccinating their children [9]. American parents also reported that other conditions, such as availability and easy access to care, were related to the decision to vaccinate their children. Furthermore, in terms of the attitude of parents toward vaccination, a study by Chen et al. applied the health benefit model to identify differences in the rate of vaccination between Caucasian children and those of other ethnic groups. The results indicated that African- American parents were hesitant regarding flu vaccination and worried that the vaccine might cause an influenza infection [21].

Most of the Saudi parents did not agree that a vaccine could cause harm to children regardless of vaccination uptake. Further qualitative research is warranted to explore which specific factors affect the decision of Saudi parents. Also, evidence shows that social support from relatives and friends is a core factor that influences the decision of parents regarding vaccination among some American parents [21]. Allison et al. also indicated that parents who were affected by the opinions of relatives and friends regarding the positive outcomes of the vaccine were more likely to vaccinate their children [22]. However, according to our findings, Saudi parents were not influenced by their relatives. It may be argued that Saudi parents did not share their decision regarding the flu vaccine with others.

Another important social support is the one provided by healthcare providers. This type of support was significantly associated with the rate of vaccination in the present and other studies [23,24]. A study by Gaglani et al. showed that a physician’s recommendation had a high impact on parents’ decisions regarding vaccination against the flu [25]. Since our study did not investigate whether physicians provided recommendations, future studies should explore whether recommendations of physicians could increase the rate of vaccination in KSA.

The perceived consequences consist of two parts: the perceived benefits of the vaccine and the perceived risks linked to non-vaccination. The perceived benefits reported in the literature were associated with parental intention to vaccinate the children. A web-based study involving Chinese parents reported that parental knowledge on the potential benefits of vaccination was positively associated with the intention to vaccinate [15]. However, this association was observed among parents with healthy children, rather than asthmatic children. Another study conducted in five European countries revealed a similar association between the perceived benefits of vaccination and the high vaccine uptake [26]. However, all reported associations were noted among healthy children. Parents with asthmatic children may hesitate to vaccinate them despite being aware of the benefits. In this study, the perceived benefits were strongly associated with vaccine uptake among asthmatic children, even after adjusting for other variables in the multivariable analysis. Parents who perceived risks in this study were more likely to be favorable toward the vaccine uptake.

## Conclusions

Although parents agreed with most of the positive statements of perception toward the vaccine against the flu in asthmatic children, the rate of vaccination among their children was low. This is one of the very few studies that has been conducted in the Middle East investigating the perception of parents about immunization against the flu in asthmatic children. The vaccination status was rechecked using the medical records of patients to avoid recall bias in the results of the questionnaire. However, our study was limited to participants from a single tertiary healthcare provider. Future research should conduct similar surveys in more facilities to get a broader understanding of the association between parental perceptions and vaccine uptake in KSA.

## Appendices

**Table 4:** Descriptive statistics of sample characteristics and parental perceptions N: number of respondents

Characteristics (n = 190)	Description	N (%)
(a) Sample characteristics		
Nationality	Saudi	185 (97.4%)
	Non-Saudi	5 (2.6%)
Age	≤20	14 (7.4%)
	21–40	133 (70%)
	>40	43 (22.6%)
Sex	Male	78 (41.1%)
	Female	112 (58.9%)
Marital status	Married	174 (91.6%)
	Divorced	2 (1.1%)
	Other	14 (7.4%)
Educational level	Not educated	10 (5.3%)
	Middle education	68 (35.8%)
	Bachelor	73 (38.4%)
	Higher education	39 (20.5%)
Occupation	Employed	100 (52.6%)
	Unemployed	78 (41.1%)
	Other	12 (6.3%)
Vaccination status	Vaccinated	88 (46.3%)
	Non-vaccinated	102 (53.7%)
(b) Perceptions		
Children with asthma should receive flu vaccination	Agree	173 (91.1%)
	Do not agree	4 (2.1%)
	Do not know	13 (6.8%)
I received a reminder from the primary care unit/hospital regarding my child’s flu vaccination appointment	Agree	137 (72.1%)
	Do not agree	25 (13.2%)
	Do not know	28 (14.7%)
It is easy to reach the primary care unit/hospital to receive the flu vaccination	Agree	166 (87.4%)
	Do not agree	14 (7.4%)
	Do not know	10 (5.3%)
I believe that my child has to receive the flu vaccination	Agree	173 (91.1%)
	Do not agree	8 (4.2%)
	Do not know	9 (4.7%)
The flu vaccination may cause complications/troubles to my child	Agree	36 (18.9%)
	Do not agree	122 (64.2%)
	Do not know	32 (16.8%)
The maximum limit of flu vaccine for children per visit is	One shot	108 (56.8%)
	Two shots	82 (43.2%)
	Three shots	0 (0.0%)
	Four shots	0 (0.0%)
I believe that my child is sick because of the flu shot	Agree	53 (27.9%)
	Do not agree	113 (59.5%)
	Do not know	24 (12.6%)
Comparing the number of times that my child got sick during last year’s winter versus this winter	Less than last winter	99 (52.1%)
	More than last winter	50 (26.3%)
	No difference	41 (21.6%)
My child’s pediatrician believes that my child should receive the flu vaccine	Agree	166 (87.4%)
	Do not agree	10 (5.3%)
	Do not know	14 (7.4%)
My relatives believe that my child should receive the flu vaccine	Agree	144 (75.8%)
	Do not agree	19 (10.0%)
	Do not know	27 (14.2%)
Non-vaccinated children are more likely to contract the flu	Agree	117 (61.6%)
	Do not agree	50 (26.3%)
	Do not know	23 (12.1%)
The flu vaccination prevents infection by the flu virus	Agree	157 (82.6%)
	Do not agree	12 (6.3%)
	Do not know	21 (11.1%)
I am worried about the chances of my child contracting the flu because of the flu vaccine	Agree	45 (23.7%)
	Do not agree	119 (62.6%)
	Do not know	26 (13.7%)

**Table 5:** Association between parental characteristics and vaccination status *Significant p-value

Characteristic	Description	Vaccinated	Non-vaccinated	P-value
Educational level	Not educated	2 (2.3%)	8 (7.8%)	<0.001*
	Middle education	20 (22.7%)	48 (47.1%)	
	Bachelor	30 (34.1%)	43 (42.2%)	
	Higher education	36 (40.9%)	3 (2.9%)	
Age	≤20	13 (14.8%)	1 (1.0%)	<0.001*
	21–40	46 (52.3%)	87 (85.3%)	
	>40	29 (33%)	14 (13.7%)	
Sex	Male	35 (39.8%)	43 (42.2%)	0.739
	Female	53 (60.2%)	59 (57.8%)	
Nationality	Saudi	87 (98.9%)	98 (96.1%)	0.232
	Non-Saudi	1 (1.1%)	4 (3.9%)	
Social status	Married	76 (86.4%)	98 (96.1%)	0.042*
	Divorced	1 (1.1%)	1 (1.0%)	
	Other	11 (12.5%)	3 (2.9%)	
Occupation	Employed	39 (44.3%)	61 (59.8%)	0.098
	Unemployed	43 (48.9%)	35 (34.3%)	
	Other	6 (6.8%)	6 (5.9%)	
Children with asthma should receive flu vaccination	Agree	77 (87.5%)	96 (94.1%)	0.224
	Do not agree	2 (2.3%)	2 (2.0%)	
	Do not know	9 (10.2%)	4 (3.9%)	
I received a reminder from the primary care unit/hospital regarding my child’s flu vaccination appointment	Agree	64 (72.7%)	73 (71.6%)	0.641
	Do not agree	13 (14.8%)	12 (11.8%)	
	Do not know	11 (12.5%)	17 (16.7%)	
It is easy to reach the primary care unit/hospital to receive the flu vaccination	Agree	69 (78.4%)	97 (95.1%)	0.002*
	Do not agree	12 (13.6%)	2 (2.0%)	
	Do not know	7 (8.0%)	3 (2.9%)	
I believe that my child has to receive the flu vaccination	Agree	78 (88.6%)	95 (93.1%)	0.533
	Do not agree	5 (5.7%)	3 (2.9%)	
	Do not know	5 (5.7%)	4 (3.9%)	
The flu vaccine may cause complications/troubles to my child	Agree	23 (26.1%)	13 (12.7%)	<0.001*
	Do not agree	42 (47.7%)	80 (78.4%)	
	Do not know	23 (26.1%)	9 (8.8%)	
The maximum limit of flu vaccine for children per visit is	One shot	83 (94.3%)	25 (24.5%)	<0.001*
	Two shots	5 (5.7%)	77 (75.5%)	
I believe that my child is sick because of the flu shot	Agree	29 (33.0%)	24 (23.5%)	0.266
	Do not agree	47 (53.4%)	66 (64.7%)	
	Do not know	12 (13.6%)	12 (11.8%)	
Comparing the number of times that my child got sick during last year’s winter versus this winter	Less than last winter	20 (22.7%)	79 (77.5%)	<0.001*
	More than last winter	46 (52.3%)	4 (3.9%)	
	No difference	22 (25.0%)	19 (18.6%)	
My child’s pediatrician believes that my child should receive the flu vaccine	Agree	72 (81.8%)	94 (92.2%)	0.016*
	Do not agree	9 (10.2%)	1 (1.0%)	
	Do not know	7 (8.0%)	7 (6.9%)	
My relatives believe that my child should receive the flu vaccine	Agree	64 (72.7%)	80 (78.4%)	0.340
	Do not agree	8 (9.1%)	11 (10.8%)	
	Do not know	16 (18.2%)	11 (10.8%)	
Non-vaccinated children are more likely to contract the flu	Agree	63 (71.6%)	54 (52.9%)	<0.001*
	Do not agree	10 (11.4%)	40 (39.2%)	
	Do not know	15 (17.0%)	8 (7.8%)	
The flue vaccination prevents infection by the flu virus	Agree	67 (76.1%)	90 (88.2%)	0.008*
	Do not agree	7 (8.0%)	5 (4.9%)	
	Do not know	14 (15.9%)	7 (6.9%)	
I am worried about the chances of my child contracting the flu because of the flu vaccine	Agree	31 (35.2%)	14 (13.7%)	0.002*
	Do not agree	46 (52.3%)	73 (71.6%)	
	Do not know	11 (12.5%)	15 (14.7%)	

**Table 6:** Independent factors associated with the flu vaccine uptake OR: odds ratio; CI: Confidence interval; *Reference range: do not know/do not agree **Significant p-value

Survey description	OR (95% CI)	P-value
It is easy to reach the primary care unit/hospital to receive the flu vaccination	0.206 (0.07–0.608)*	*0.004**
Non-vaccinated children are more likely to contract the flu	2.479 (1.200–5.119)*	*0.014**
The flu vaccination prevents infection by the flu	0.395 (0.157–0.992)*	*0.048**
I am worried about the chances of my child contracting the flu because of the flu vaccine	2.913 (1.367–6.208)*	*0.006**
